# Isotope-Substitution Effects on the Thermodynamic, Dynamic, and Structural Properties of Water: H_2_O, HDO, D_2_O, and T_2_O

**DOI:** 10.1021/acs.jpcb.5c01657

**Published:** 2025-06-29

**Authors:** Ali Eltareb, Gustavo E. Lopez, Nicolas Giovambattista

**Affiliations:** † Department of Physics, 2037 Brooklyn College of the City University of New York, Brooklyn, New York 11210, United States; ‡ Ph.D. Program in Physics, 14772 The Graduate Center of the City University of New York, New York, New York 10016, United States; ¶ Department of Chemistry, Lehman College of the City University of New York, Bronx, New York 10468, United States; § Ph.D. Program in Chemistry, 14772 The Graduate Center of the City University of New York, New York, New York 10016, United States

## Abstract

We study the isotope-substitution effects on the thermodynamic, dynamical, and structural properties of liquid water at (i) constant molar volume (*v* = 18.0 cm^3^/mol, corresponding to a density for H_2_O of ρ = 1.0 g/cm^3^) and (ii) constant pressure (*P* = 0.1 MPa) over a wide temperature range, 200 ≤ *T* ≤ 400 K. Our results are based on path-integral and classical computer simulations of H_2_O, HDO, D_2_O, and T_2_O using the q-TIP4P/F water model. We find that some properties, such as the pressure *P*(*T*) (at constant *v*) and molar volume *v*(*T*) (at constant *P*) are weakly sensitive to isotope substitution effects, while others, including the isochoric/isobaric heat capacity, self-diffusion coefficient, vibrational density of states, and infrared (IR) spectra, are considerably affected by nuclear quantum effects (NQE). The IR spectra and diffusion coefficients obtained from ring-polymer molecular dynamics (RPMD) simulations are in very good agreement with available experimental data. Our path integral computer simulations, particularly at low temperatures, show that the (H → D → T)-substitution in water leads to a slightly *more structured* liquid with shorter (smaller OO distance) and more linear (smaller HOO angle) hydrogen bonds (HB). This is rationalized in terms of the very small *decrease* in the atom delocalization (NQE) along the sequence (H → D → T). In all three cases, the H/D/T atoms are preferentially delocalized along the direction perpendicular to the O-(H/D/T) covalent bond. The different delocalization of H/D/T leads to a *slightly* more energetic HB (<4%) and hence, to a *slightly* stronger HB-network, along the sequence H_2_O → HDO → D_2_O → T_2_O (as NQE becomes less pronounced). Interestingly, some properties of HDO, such as the IR spectra, radial distribution functions, and HB geometry, suggest that the OD and OH covalent bonds of HDO behave, respectively, as the OD covalent bond of D_2_O and the OH covalent bond of H_2_O.

## Graphical abstract

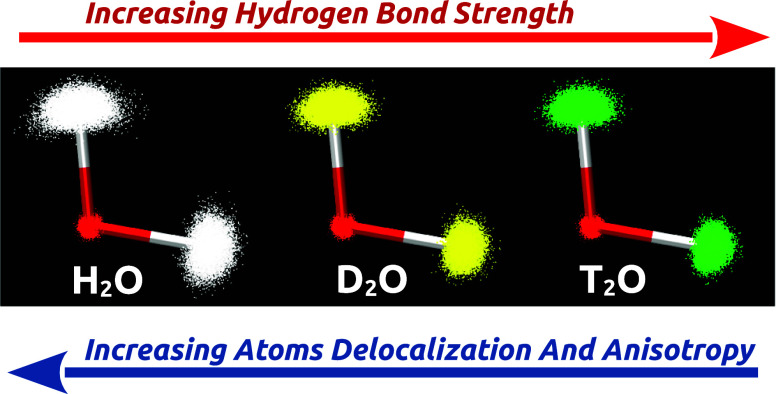

## Introduction

Water plays a fundamental role across a wide range of scientific disciplines, including astrophysics,^1^ atmospheric chemistry,^2^ biology,^3–5^ biochemistry,^6,7^ and chemical/biological/materials engineering.^8^ Accordingly, it is not surprising that the properties of water have been the focus of numerous studies for centuries.^9,10^ It has been shown that water exhibits a wide range of thermodynamic and dynamic anomalous properties that set it apart from most liquids.^11–14^ For example, water exhibits an anomalous density maximum at 277 K (*P* = 0.1 MPa), which allows ice to float on liquid water, supporting aquatic life at subfreezing temperatures.^9^ Water also has a very large heat capacity and surface tension, which play vital roles in temperature regulation in the human body and enable capillary action in plants.^15^ Importantly, the anomalous behavior of liquid water becomes more pronounced at very low temperature, in the supercooled liquid state (*T* < 273 K at 1 bar).^14,16^

The anomalous properties of water are not limited to H_2_O but are also observed in its isotopes, including D_2_O and T_2_O.^17^ However, isotope-substitution effects lead to small temperature shifts in the properties of water. For example, at *P* = 0.1 MPa, the melting temperature *T*_M_ of H_2_O is 5 K lower than the *T*_M_ of D_2_O, and the *T*_M_ of D_2_O is 3 K lower than the *T*_M_ of T_2_O.^9,17^ We note that the corresponding temperature shifts δ*T* vary among the properties considered. For example, the temperature of maximum density and maximum isothermal compressibility of H_2_O and D_2_O differ by δ*T* = 7.2 and 4 K, respectively.^17–20^ The dynamical properties of water are also affected by isotope substitutions; for example, the glass transition temperature *T*_g_ of H_2_O is approximately 10 K lower than the *T*_g_ of D_2_O.^21^ These different temperature shifts in the properties of the water isotopes demonstrate that the corresponding nuclear quantum effects (NQE) cannot be captured by simple temperature-independent scaling laws.^22,23^

Understanding the isotope-substitution effects on the properties and phase behavior of water, while being of fundamental scientific relevance, is important for practical applications.^24–26^ In many experimental techniques, including infrared and nuclear magnetic-resonance spectroscopy, D_2_O is commonly used as a solvent instead of H_2_O. This is because the signal generated by H_2_O in these experiments may interfere with the corresponding signal generated by the sample being studied. For example, in the case of infrared spectroscopy, the vibrational mode frequencies of H_2_O (but not of D_2_O) may overlap with the vibrational mode frequencies of specific protein groups.^27–29^ In these applications, the differences in the thermodynamic and dynamic properties of D_2_O and H_2_O, as well as the subtle differences in the corresponding hydrogen-bond (HB) network and HB strength, are usually ignored. However, such subtle differences in water may affect the sample being studied.^30^ In biological systems, the choice of H_2_O or D_2_O can significantly influence the properties of protein solutions.^30–33^ The difference between H_2_O and D_2_O can affect protein structure and stability, making it essential to examine the isotope substitution effects in water.^30^

In this work, we study the isotope-substitution effects on the thermodynamic, dynamic, and structural properties of water by performing path-integral computer simulations of H_2_O, HDO, D_2_O, and T_2_O. We cover a wide range of temperatures, focusing on the low-temperature supercooled liquid states where the anomalous properties of water, as well as the NQE, become more pronounced. Our results reveal that isotope-substitution effects are minor on some properties, such as the pressure *P*(*T*) at constant molar volume and the molar volume *v*(*T*) at constant pressure, as well as the average water structure. Instead, other properties, such as the isobaric and isochoric heat capacities, self-diffusion coefficient, vibrational density of states, and infrared (IR) spectra, are considerably affected by NQE. We show that, along the sequence H_2_O → HDO → D_2_O → T_2_O and particularly, at low temperatures, (i) the HB network of water becomes slightly more tetrahedral and stronger, and (ii) with shorter, more linear, and more energetic HB. Ultimately, these changes are correlated with a decreasing atom delocalization along the H → D → T substitution.

This work is organized as follows. We first present the computer simulation details and then discuss the results of our computer simulations for H_2_O, HDO, D_2_O, and T_2_O using the q-TIP4P/F water model. A summary and discussion are included in the last section of this work.

## Simulation Method

We perform ring-polymer molecular dynamics (RPMD) simulations of H_2_O, HDO, D_2_O, and T_2_O using the q-TIP4P/F water model (RPMD reduces to path-integral molecular dynamics (PIMD) simulations when thermodynamics and structural properties are considered).^34^ This is a flexible water model, where the OH covalent bond potential energy is represented with a quartic expansion of a Morse potential, and the HOH angle potential energy is represented by a simple harmonic potential. In the case of HDO, all water molecules are modeled as singly deuterated, i.e., consisting of one hydrogen and one deuterium atom per molecule (100% HDO). We note that this is a model system that is not experimentally realizable due to rapid H/D exchange in liquid water,^35^ but it provides a useful intermediate model system, between H_2_O and D_2_O, for the study of isotope substitution effects in water. Previous computational studies show that the q-TIP4P/F water model reproduces remarkably well many of the properties of liquid water,^34,36,37^ ice I_h_,^38–40^ and glassy water (LDA and HDA)^38,41,42^ at *P* = 0.1 MPa.

Computer simulations are performed at (i) constant volume (*v* = 18.0 cm^3^/mol; corresponding to a density for H_2_O of ρ = 1.0 g/cm^3^) for temperatures 200 ≤ *T* ≤ 400 K, and (ii) constant pressure (*P* = 0.1 MPa) for temperatures 220 ≤ *T* ≤ 400 K. The system is composed of *N* = 512 water molecules placed in a cubic box (side length *L* = 2.4837 nm for *v* = 18.0 cm^3^/mol) with periodic boundary conditions along all three directions. Our computer simulations at *P* = 0.1 MPa are motivated by the fact that most experiments on water isotopes are typically performed at this pressure. Our computer simulations at constant molar volume are meant to expose the role of isotope substitution effects/NQE without the additional effects due to volume differences among the water isotopes (at a given pressure) and the density fluctuations induced by an external barostat, which may vary from isotope to isotope. Such isotope-dependent volume and volume fluctuations (at a given pressure) may, in principle, alter the structural and dynamical properties of water to a different degree.

We follow the same computational techniques employed in our previous studies^36,37^ and refer the reader to those studies for details. Briefly, we control the temperature of the system using a stochastic (local) path-integral Langevin equation (PILE) thermostat^43^ where the thermostat collision frequency parameter is set to γ = 0.1 ps^–1^. In the constant pressure simulations, the pressure of the system is maintained by using a Monte Carlo barostat.^44^ Short-range (Lennard-Jones pair potential) interactions are calculated using a cutoff of *r*_c_ = 1.0 nm, and the long-range electrostatic interactions are computed using the reaction-field technique^45^ with the same cutoff *r*_c_. In the reaction-field calculations, the dielectric constant (relative permittivity) of the continuum medium beyond the cutoff radius *r*_c_ is set to 78.3. In the RPMD/PIMD simulations, the time step is *dt* = 0.25 fs, and the number of beads per ring-polymer/atom is set to *n*_b_ = 32. As shown in previous studies, most of the relevant thermodynamic, dynamical, and structural properties of q-TIP4P/F water calculated from RPMD/PIMD simulations are converged for *n*_b_ = 32.^36,37^ These include the density, isothermal compressibility, thermal expansion coefficient, radial distribution functions, and diffusion coefficient of H_2_O at approximately *T* > 200 K and 1 bar. However, the enthalpies and heat capacities (*C*_P_ and *C*_V_) may differ by <1 kJ/mol and 5–30 J/(mol K), respectively, as *n*_b_ varies in the range *n*_b_ = 32–128.^36,37^ In this regard, we note that our results for Cp and Cv, Figures 1e and 2c, should be taken with caution and only as indicative of the expected trend in the corresponding isotope substitution effects; it would be important in the future to calculate *C*_P_ and *C*_V_ using advanced techniques, such as projected Hessians or higher order Trotter factorization schemes,^46–48^ to provide a more accurate estimate of the NQE on the *C*_P_ and *C*_V_ of water. For comparison, we also performed classical MD simulations of H_2_O. This is done by performing PIMD simulations with *n*_b_ = 1. The same computational details described above hold for the classical MD simulations, except that the time step is increased to *dt* = 0.5 fs. At each temperature, the system is equilibrated for 1–50 ns, depending on the temperature. Equilibration runs are followed by production runs where computer simulations are performed for an additional 1–100 ns (depending on the temperature); see also refs 36 and 37. All the PIMD/RPMD simulations are performed using the OpenMM (version 7.5.0) software package.^49^

**Figure 1:**
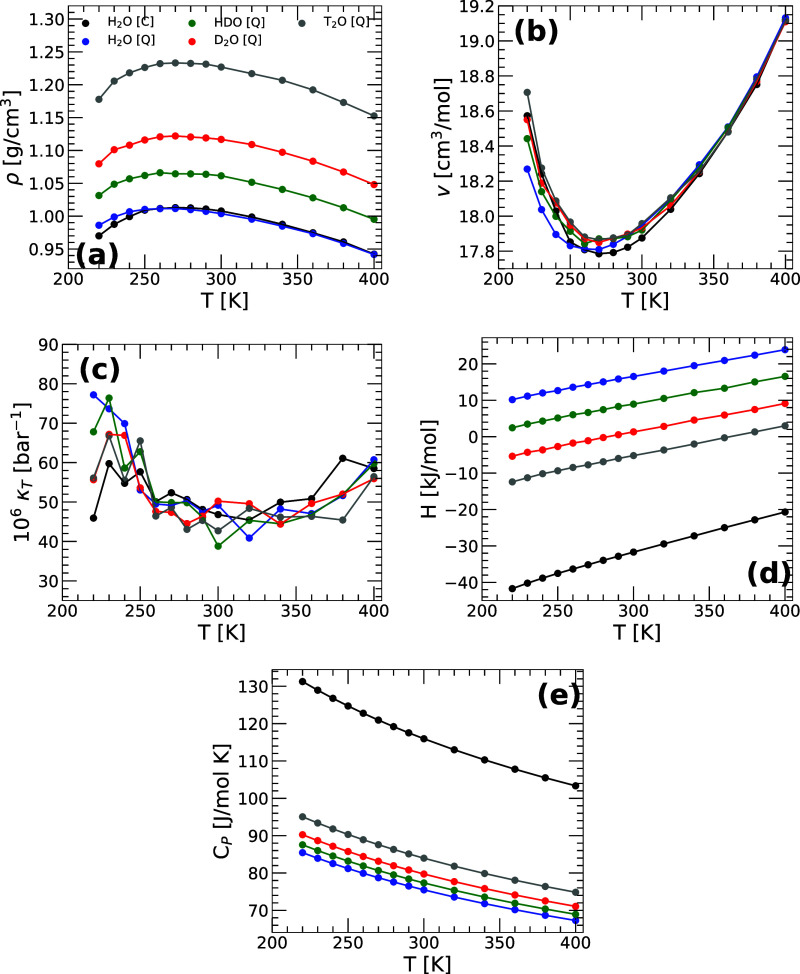
(a) Density ρ­(*T*), (b) molar volume *v*(*T*), (c) isothermal compressibility κ_*T*_(*T*), (d) enthalpy *H*(*T*), and (e) isobaric heat capacity *C*_P_(*T*) as a function of temperature for the isotopes of q-TIP4P/F water obtained from PIMD simulations at *P* = 0.1 MPa. For comparison, results from classical MD simulations of H_2_O are also included and are indicated by black solid circles. The isobaric heat capacity *C*_P_(*T*) in (e) was calculated by fitting the enthalpies in (d) to a fourth-order polynomial.

**Figure 2:**
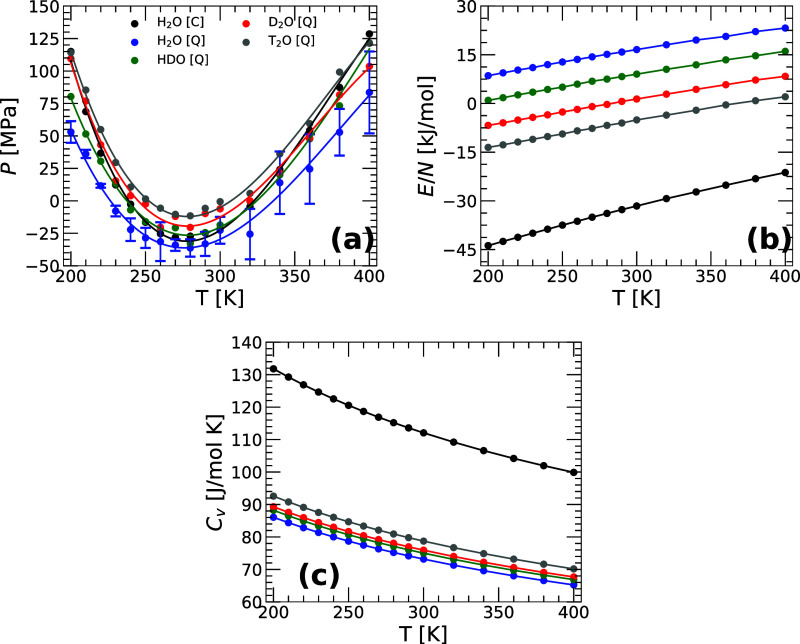
(a) Pressure (b) total energy, and (c) isochoric heat capacity *C*_V_(*T*) as a function of temperature from PIMD simulations of q-TIP4P/F water (*v* = 18.0 cm^3^/mol; ρ = 1.0 g/cm^3^ for H_2_O). Results for H_2_O, HDO, D_2_O, and T_2_O are indicated by blue, green, red, and gray lines, respectively. For comparison, also included are the results for H_2_O (black line) obtained from classical MD simulations. The *P*(*T*) values for the different water isotopes are within a range of 25–75 MPa, which is of the order of the error bars in *P*(*T*), [δ*P* ≈ 25 MPa; error bars are shown for the case of H_2_O (blue) only]. In all cases, a minimum in *P*(*T*) occurs at *T* ≈ 270–280 K implying that all water isotopes exhibit a density maximum upon isobaric cooling (see the text). At a given temperature, the values of *E*(*T*) and *C*_V_(*T*) vary monotonically [*E*(*T*) increases while *C*_V_(*T*) decreases] along the sequence H_2_O (classical) → T_2_O → D_2_O → HDO → H_2_O (quantum).

## Results

The results are organized as follows. We first discuss the thermodynamic (volume and pressure, isobaric/isochoric specific heat, and enthalpy/energy) and dynamical properties (diffusion coefficient, vibrational density of states, and IR spectra) of H_2_O, HDO, D_2_O, and T_2_O. Then the structure (radial distribution functions and local order) of the target water isotopes is studied. We conclude with a discussion of the HB properties (OO length, HOO angle, HB energy) of H_2_O, HDO, D_2_O, and T_2_O and the corresponding NQE due to the H/D/T atom delocalization.

### Thermodynamic Properties

#### Density, Enthalpy, and Isobaric Heat Capacity

We discuss first the results from computer simulations at *P* = 0.1 MPa. Figure 1 shows the density ρ­(*T*), molar volume *v*(*T*), isothermal compressibility κ_*T*_(*T*), enthalpy *H*(*T*), and isobaric heat capacity *C*_P_(*T*) of H_2_O, HDO, D_2_O, and T_2_O as a function of temperature at *P* = 0.1 MPa. For comparison, classical MD simulation results for H_2_O are included and are indicated by black solid circles.

As shown in Figure 1, all isotopes of q-TIP4P/F water exhibit an anomalous density maximum, ρ_max_, at temperatures *T*_max_^ρ^ ≈ 270 – 280 K at *P* = 0.1 MPa. The corresponding temperatures *T*_max_^ρ^ obtained from MD/PIMD simulations are summarized in Table 1 along with the corresponding experimental values at *P* = 0.1 MPa. Our values of *T*_max_^ρ^ are close to the experimental values reported at *P* = 0.1 MPa^9,50–52^ but the corresponding deviations are approximately Δ*T* ≈ 5–13 K depending on the isotope considered. We note that the experimental values of *T*_max_^ρ^ at *P* = 0.1 MPa increase slightly as the mass of the isotope increases. However, within the precision of our PIMD simulations, we cannot conclusively demonstrate that the density maximum indeed shifts with an increase in isotope mass. Notably, the density of H_2_O from PIMD simulations overlaps with that obtained from classical MD simulations, suggesting that the inclusion of NQE does not significantly influence the densities of liquid q-TIP4P/F water (*T* ≥ 240 K) (this is consistent with refs 36 and 37 using the same water model employed here but treating the long-range electrostatic interactions using the Particle Mesh Ewald (PME) method). Interestingly, the very different densities of the water isotopes studied correspond to small changes in the corresponding molar volumes (up to 1–2% for *T* = 240 K). As shown in Figure 1b, the molar volumes of H_2_O, HDO, D_2_O, and T_2_O overlap at approximately *T* > 350 K, as expected since NQE should be negligible at high temperatures. Instead, at low temperatures, the molar volume of the isotopes studied varies slightly, particularly as the temperature decreases.

**Table 1:** Temperature of Maximum Density, *T*_max_^ρ^ for H_2_O, HDO, D_2_O, and T_2_O At *P* = 0.1 MPa, Obtained from PIMD [Q] and Classical MD [C] Simulations, As Well As Experiments;^9,50–52^ See Also Figure[Fig fig1]a^a^

isotope	PIMD/MD *T*_max_^ρ^ (K)	exp. *T*_max_^ρ^ (K)	PIMD/MD *C*_P_ (J/mol K)	exp. *C*_P_ (J/mol K)
H_2_O [Q]	267 (3)	277	75	75
HDO [Q]	272 (2)	277	77	
D_2_O [Q]	272 (2)	284	80	84
T_2_O [Q]	273 (2)	286	84	
H_2_O [C]	272 (2)	277	115	74

a Also included are the values for the isobaric heat capacity *C*_P_(*T*), at *T* = 300 K (*P* = 0.1 MPa) from PIMD/MD simulations and experiments.^54,56^ Numbers in parentheses are standard deviations.

The isothermal compressibility κ_T_(*T*) shown in Figure 1c follows the expected experimental trend for all the water isotopes, where isobaric cooling results in an increased compressibility^18,52–54^ at low temperatures, and a minimum develops at high temperatures. Our PIMD simulations indicate that κ_T_(*T*) are rather identical (within error bars) among the H_2_O, HDO, D_2_O, and T_2_O. This implies that the inclusion of NQE does not substantially affect the volume fluctuations of q-TIP4P/F water, at least at 0.1 MPa.

We stress that not all of the thermodynamic properties of water are insensitive to isotope substitution effects. To show this, included in Figure 1d,e are the enthalpy and isobaric heat capacity of the water isotopes studied. Our MD/PIMD simulations show that as the mass of the water isotope increases, the enthalpy *H*(*T*) decreases while the isobaric heat capacity *C*_P_(*T*) increases. The increase in *C*_P_(*T*) upon isobaric cooling is consistent with experiments of H_2_O and D_2_O.^55^ Similarly, also consistent with experiments,^56^ we find that at a given temperature (*T* ≥ 240 K), the *C*_P_(*T*) increases along the sequence H_2_O → HDO → D_2_O → T_2_O. Our results in Figure 1e (and Figure 2c, see below) are based on path integral computer simulations using nb=32 beads per ring-polymer and hence, they should be taken with caution and only as indicative of the expected trend in the isotope substitution effects on the heat capacities of q-TIP4P/F water (see Simulations Details).

#### Pressure, Energy, and Isochoric Heat Capacity

Next, we discuss the thermodynamic properties of the water isotopes at a constant molar volume. Figure 2 shows the (a) pressure *P*(*T*), (b) total energy *E*(*T*), and (c) isochoric heat capacity *C*_V_(*T*) = (∂*E*/∂*T*)_V_ as a function of temperature for the different water isotopes obtained from PIMD simulations at *v* = 18.0 cm^3^/mol. For comparison, the corresponding values from classical MD simulations of H_2_O are also included. As shown in Figure 2a, in all cases, *P*(*T*) exhibits a minimum at approximately, *T* ≈ 270–280 K. It can be shown that the presence of a minimum in *P*(*T*) at constant volume implies that the liquid exhibits an anomalous density maximum upon isobaric cooling [at the pressure corresponding to the minimum in *P*(*T*)].^57–59^ Accordingly, the behavior of *P*(*T*) in Figure 2a is fully consistent with the maximum density shown in Figure 1a. Note that the differences in *P*(*T*) among the different water isotopes are within a range of 25–75 MPa, which is of the order of the error bars in *P*(*T*) (δ*P* ≈ 25 MPa).

The total energy *E*(*T*) of the different water isotopes is shown in Figure 2b. As for the case of *H*(*T*) in Figure 1d, at a given temperature, *E*(*T*) increases monotonically along the sequence H_2_O­(classical) → T_2_O → D_2_O → HDO → H_2_O, i.e., as the NQE becomes more pronounced and the H isotope becomes more delocalized (see Section 3.4). This trend in the total energy is expected since PIMD simulations incorporate NQE, such as zero-point energy (ZPE), which significantly contributes to the vibrational modes associated with bond stretching and angle bending. The energy contributions from these modes are dependent on the mass of the H isotope. H_2_O, with its lighter hydrogen atom, has larger vibrational mode frequencies than D_2_O and T_2_O. Consequently, the energies of the water isotopes obtained from the PIMD simulations decrease along the sequence H_2_O (quantum) → HDO → D_2_O → T_2_O. Similarly, since the ZPE is excluded in classical MD simulations, the energy values of H_2_O obtained from PIMD simulations are larger than those obtained from classical MD simulations of H_2_O. Our results highlight the importance of including NQE. For example, the energy difference between T_2_O and H_2_O remains substantial at all temperatures, approximately 20–25 kJ/mol, comparable to the energy of the HB (≈20 kJ/mol). In addition to the contribution of the ZPE to *E*(*T*), it could be possible that the different values of *E*(*T*) among the water isotopes are also due to the variations in the HB energy due to the H/D/T substitution. As shown in Section 3.4, the energy/strength of the HB is indeed different in H_2_O, HDO, D_2_O, and T_2_O. However, these energy contributions to *E*(*T*) are small and hence, the main changes in the *E*(*T*) shown in Figure 1c are due to ZPE.

Figure 2c shows the isochoric heat capacity *C*_V_(*T*) for all of the water isotopes considered. *C*_V_(*T*) is calculated by fitting the total energies *E*(*T*) shown in Figure 2b to a fourth-order polynomial, followed by taking the corresponding derivative with respect to *T*, i.e., *C*_V_(*T*) ≡ (∂*E*/∂*T*)_V_. As for the case of *C*_P_(*T*) in Figure 1e, *C*_V_(*T*) increases upon cooling, implying that the energy fluctuations in the corresponding water isotopes increase anomalously with decreasing temperature. It follows that the behavior of *C*_V_(*T*) is anomalous for all isotopes since the energy fluctuations in normal liquids decrease upon cooling. Interestingly, at a given temperature, *C*_V_(*T*) (and hence, the energy fluctuations) decreases monotonically along the sequence H_2_O­(classical) → T_2_O → D_2_O → HDO → H_2_O, i.e., as the NQE becomes more pronounced. For example, at *T* < 300 K, the values of *C*_V_(*T*) for T_2_O are 5–10 J/mol/K (≈10%) larger than those for H_2_O. It follows that, consistent with previous PIMD computational studies,^36,60^ the inclusion of NQE is crucial for an accurate evaluation of *C*_V_(*T*).

### Dynamics and Vibrational Density of States

#### Diffusion Coefficient

We calculated the self-diffusion coefficient *D*(*T*) of the target water isotopes from RPMD simulations of q-TIP4P/F water. *D*(*T*) is obtained by using the same technique described in previous studies.^36,37,61,62^ Briefly, the diffusion coefficient is derived from the mean-square displacement (MSD) of the ring-polymers' centroids (center of mass) associated with the water O atoms; *D*(*T*) is evaluated from the slope of the MSD­(*t*) as a function of time, for long times at which MSD­(*t*) = 6*Dt*.

The values of *D*(*T*) at *P* = 0.1 MPa and *v* = 18.0 cm^3^/mol are shown in Figure 3a (squares and circles, respectively). At a given temperature, the values of *D*(*T*) obtained at *P* = 0.1 MPa and *v* = 18.0 cm^3^/mol practically overlap. Accordingly, next, we will focus on the results obtained at constant *v* = 18.0 cm^3^/mol (circles in Figure[Fig fig3]b–d). At high temperatures, *T* ≥ 300 K, the system has sufficient kinetic energy to overcome potential energy barriers. In this regime, the system is highly diffusive, and the diffusion coefficient of the water isotopes obeys the Arrhenius equation, i.e.,

$$D(T)=D_{0}\exp(-E_{A}/k_{B}T)$$ 1

where *D*_0_ and *E*_A_ are constants, and *k*_B_ is the Boltzmann constant. Figure 3b displays the values of *D*(*T*) for all of the water isotopes studied and shows that eq 1 holds in all cases for *T* ≥ 300 K. The values of *E*_A_ and *D*_0_ are given in Table 2. The values of *D*_0_ are rather similar for all of the water isotopes. Instead, the activation energies, *E*_A_, decrease along the sequence T_2_O → D_2_O → HDO → H_2_O, implying that the NQE lowers the potential energy barriers of water. While the fitting parameters *E*_A_ and *D*_0_ may depend slightly on the T-range considered to fit the data, Table 2 suggests that, at *v* = 18.0 cm^3^/mol, the heavier isotopes of q-TIP4P/F water require slightly more energy (larger *E*_A_) to surmount potential energy barriers during diffusion.

**Figure 3:**
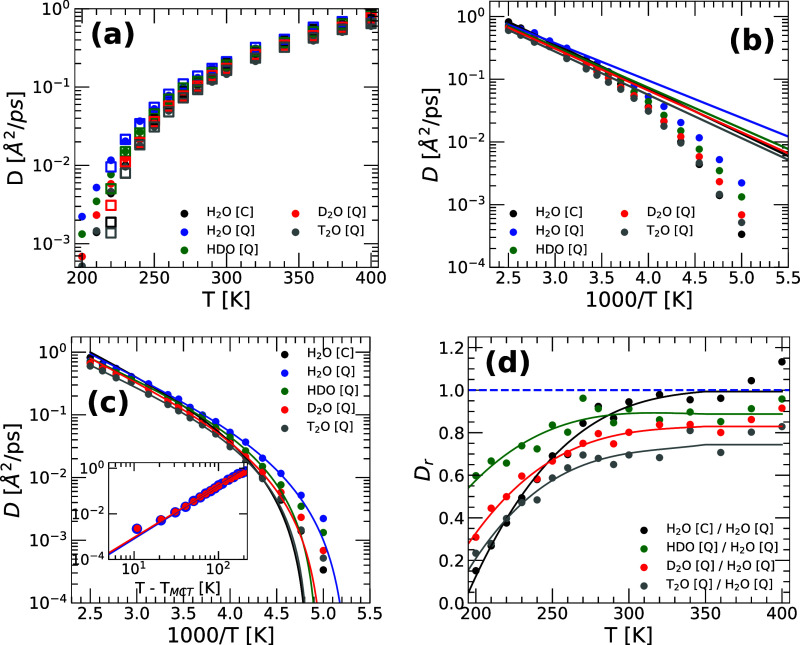
(a) Diffusion coefficient of water isotopes obtained from RPMD simulations of q-TIP4P/F water at *P* = 0.1 MPa (squares) and *v* = 18.0 cm^3^/mol (circles). (b) *D*(*T*) at *v* = 18.0 cm^3^/mol [from (a)] together with the fit to an Arrhenius law for 300 ≤ *T* ≤ 400 K (eq 1). A dynamical crossover from the Arrhenius regime to a non-Arrhenius regime occurs at *T*_*x*_ ≈ 250 K for all water isotopes. (c) At low temperatures, the *D*(*T*) for all the water isotopes is well described by the MCT equation (eq 2). Lines are the fit to *D*(*T*) using eq 2 for 220 ≤ *T* ≤ 300 K. The inset shows the values of *D* as a function of (*T* – *T*_MCT_) in a log–log scale for H_2_O and D_2_O obtained from the RPMD simulations (*T*_MCT_ is the MCT temperature; see Table 3). (d) Relative diffusion coefficient *D*_r_ of HDO, D_2_O, and T_2_O from RPMD simulations at *v* = 18.0 cm^3^/mol. *D*_r_ is the ratio of the values of *D*(*T*) shown in (a) to the values of *D*(*T*) for H_2_O obtained from the RPMD simulations (lines are guide-to-the-eye). As expected, *D*_r_(*T*) < 1 for all water isotopes, i.e., isotope substitution decreases the mobility of water, particularly at low temperatures.

**Table 2:** Fitting Parameters Defined in Eq 1 (Arrhenius Equation) for the Diffusion Coefficient of the Studied Water Isotopes^a^

	*v* = 18.0 cm^3^/mol (NVT)		*P* = 0.1 MPa (NPT)	
isotope	*E*_A_ (kJ/mol)	*D*_0_ (Å/ps^2^)	*E*_A_ (kJ/mol)	*D*_0_ (Å/ps^2^)
H_2_O [Q]	11.4 (0.8)	23.0 (6.2)	12.0 (1.0)	29.5 (9.9)
HDO [Q]	12.5 (0.3)	29.9 (2.5)	14.0 (0.3)	53.3 (4.6)
D_2_O [Q]	12.8 (0.4)	31.3 (3.8)	14.7 (0.4)	63.5 (8.7)
T_2_O [Q]	13.2 (0.7)	32.4 (7.9)	13.5 (0.3)	37.9 (4.2)
H_2_O [C]	13.7 (0.3)	49.8 (5.4)	14.3 (0.4)	62.3 (7.5)

a Figure 3b shows the values of *D*(*T*) for H_2_O, HDO, D_2_O, and T_2_O obtained from classical MD and RPMD simulations together with the corresponding fit using eq 1 for *T* ≥ 300 K. Numbers in parentheses are standard errors of the fitting parameters.

Consistent with previous classical MD simulations of various rigid water models,^63–65^ Figure 3b shows that *D*(*T*) evolves from an Arrhenius regime at high temperature, to a non-Arrhenius regime at low temperatures. The dynamical crossover temperature is *T*_*x*_ ≈ 250 K for all water isotopes. At *T* < *T*_*x*_, the behavior of *D*(*T*) is well-described by the mode coupling theory (MCT) prediction,^63,64,66,67^

$$D(T)=D_{1}(T-T_{\mathrm{MCT}}{)}^{\gamma }$$ 2

where *D*_1_ and γ are constants, and *T*_MCT_ is the MCT temperature. Figure 3c shows the fit to *D*(*T*) using eq 2 for the temperature range 220 ≤ *T* ≤ 300 K. The fitting parameters γ and *T*_MCT_ for water and its isotopes, obtained from our RPMD simulations at *v* = 18.0 cm^3^/mol, are given in Table 3. For comparison, also included in Table 3 are the values of γ and *T*_MCT_ obtained from our RPMD simulations at *P* = 0.1 MPa as well as the experimental values at the same pressure. The values of γ and *T*_MCT_ from RPMD simulations and experiments are relatively close to one another. The values of γ and *T*_MCT_ in Table 3 are sensitive to the details of the fitting procedure, which makes it difficult to make quantitative conclusions. The values of γ and *T*_MCT_ from MD/RPMD simulations in Table 3 are close to those obtained in experiments, but they exhibit a nonmonotonic behavior among the isotopes, which is probably due to the uncertainty in these values. The experimental values in Table 3 suggest that *T*_MCT_ increases as the water isotope mass increases, which is consistent with the corresponding decrease in the diffusivity.

**Table 3:** Fitting Parameters Defined in Eq 2 (MCT Prediction) for the Diffusion Coefficient of H_2_O, HDO, D_2_O, and T_2_O at *v* = 18.0 cm^3^/mol and *P* = 0.1 MPa^a^

	*v* = 18.0 cm^3^/mol (NVT)		*P* = 0.1 MPa (NPT)		exp.	
isotope	*T*_MCT_ (K)	γ	*T*_MCT_ (K)	γ	*T*_MCT_ (K)	γ
H_2_O [Q]	189 (12)	2.2 (0.4)	211 (3)	1.6 (0.1)	213 (3)	2.1 (0.2)
HDO [Q]	202 (6)	2.0 (0.2)	211 (10)	1.7 (0.3)		
D_2_O [Q]	199 (4)	2.3 (0.1)	207 (10)	2.0 (0.3)	225 (5)	1.6 (0.2)
T_2_O [Q]	206 (4)	2.0 (0.1)	198 (12)	2.4 (0.4)		
H_2_O [C]	206 (6)	2.2 (0.2)	209 (7)	2.1 (0.2)	213 (3)	2.1 (0.2)

a The parameters at *v* = 18.0 cm^3^/mol are obtained by fitting *D*(*T*) for 220 ≤ *T* ≤ 300 K using eq 2 (see Figure 3c). Similarly, the parameters for *P* = 0.1 MPa are obtained by fitting *D*(*T*) using the temperature interval 240 ≤ *T* ≤ 300 K. For comparison, we also include the experimental values of γ and *T*_MCT_ for H_2_O and D_2_O at *P* = 0.1 MPa [we calculate γ and *T*_MCT_ by fitting the values of *D*(*T*) reported in the experiments of ref 68 at *P* = 0.1 MPa and (240 ≤ *T* ≤ 280 K) using eq 2]. Numbers in parentheses are standard errors to the fitting parameters.

To compare the diffusivity of the different water isotopes, we show in Figure 3d the relative diffusion coefficients *D*_r_ of HDO, D_2_O, and T_2_O. *D*_r_ is the ratio of *D*(*T*) of the different isotopes (Figure 3a) to the value of *D*(*T*) for H_2_O obtained from the RPMD simulations. We find that *D*_r_ < 1 for all temperatures and all isotopes considered, indicating, again, that (quantum) H_2_O diffuses more rapidly than the heavier isotopes. The effects become particularly pronounced at lower temperatures; for example, at *T* = 200 K, the diffusivity of T_2_O is only ≈20% that of (quantum) H_2_O. Figure 3d also shows that the role of NQE is particularly important for H_2_O at low temperatures. At *T* = 200 K, classical water (black line) has a diffusion coefficient that is only ≈10% that of (quantum) H_2_O (blue line). This is consistent with previous PIMD simulations of H_2_O at normal pressure based on the same water model but using the PME technique to treat electrostatic interactions.^34,36,37^

#### Vibrational Density of States and Infrared Spectra

The vibrational density of states (VDOS) of the different isotopes of water are calculated by taking the Fourier transform of the Kubo-transformed velocity autocorrelation function of all the water atoms in the system (see also refs 34 and 69–71 and Supporting Information (SI)).

Given the similarities in the *D*(*T*) evaluated at *P* = 0.1 MPa and *v* = 18.0 cm^3^/mol (see Figure 3), in this section, we focus on the results obtained from the RPMD simulations at *v* = 18.0 cm^3^/mol. Figure 4a–c shows the VDOS of H_2_O, HDO, D_2_O, and T_2_O at *T* = 240 K (solid lines). The VDOS is divided into three regions corresponding to the (i) translational and librational modes (ω < 800–1000 cm^–1^, Figure 4a), (ii) bending modes (800–1000 cm^–1^ < ω < 1800 cm^–1^, Figure 4b), and (iii) stretching modes (ω > 1800 cm^–1^, Figure 4c).

i. In the low-frequency region of the VDOS (Figure 4a), increasing the mass of the isotope causes a red shift in the spectra, with the VDOS shifting toward lower frequencies along the sequence H_2_O → HDO → D_2_O → T_2_O. This leads to an overlap of the librational and translational mode frequencies. For example, in the case of (quantum) H_2_O (blue solid line), the translational and librational mode frequencies correspond to the region ω < 375 cm^–1^ and ω > 375 cm^–1^ in Figure 4a. Instead, in the case of T_2_O, the translational and librational mode frequencies cannot be distinguished.
  For comparison, also included in Figure 4a are the VDOS of each water isotope obtained from classical MD simulations (dashed lines). Notably, the inclusion of NQE barely affects the shape of the VDOS at low frequencies and the location of the corresponding peaks (the solid and dashed lines in Figure 4a practically overlap with one another).
ii. Figure 4b shows the VDOS corresponding to the bending modes from RPMD (solid lines) and MD simulations (dashed lines). The bending modes shift to lower frequencies as the mass of the isotope increases. For instance, the bending mode frequency decreases by δω ≈ 600 cm^–1^ when going from H_2_O to T_2_O.
  Interestingly, a comparison of the VDOS from classical MD (dashed lines) and PIMD simulations (solid lines) shows a shift of about δω ≈ −25 – 50 cm^–1^. The inclusion of NQE (solid lines) induces small but noticeable red shifts of the bending modes for all the isotopes considered. The effect of NQE is particularly pronounced for H_2_O (solid and dashed blue lines) and becomes less significant for the heavier isotopes, such as T_2_O, for which the red shift in the bending modes is minimal (solid and dashed gray lines).
iii. Figure 4c shows the VDOS corresponding to the stretching modes. As for the bending modes (Figure 4b), the stretching modes also shift toward lower frequencies as the isotope mass increases. The inclusion of NQE is also noticeable in the stretching mode region of the VDOS for all the isotopes considered. For example, in the case of H_2_O, including NQE shifts the stretching mode peak by δω ≈ −70 cm^–1^ (solid and dashed blue lines); δω decreases as the isotope mass increases with δω ≈ −25 cm^–1^ for T_2_O (solid and dashed gray lines). Interestingly, in the case of T_2_O, classical MD simulations show a clear double stretching mode peak (dashed gray line).

**Figure 4:**
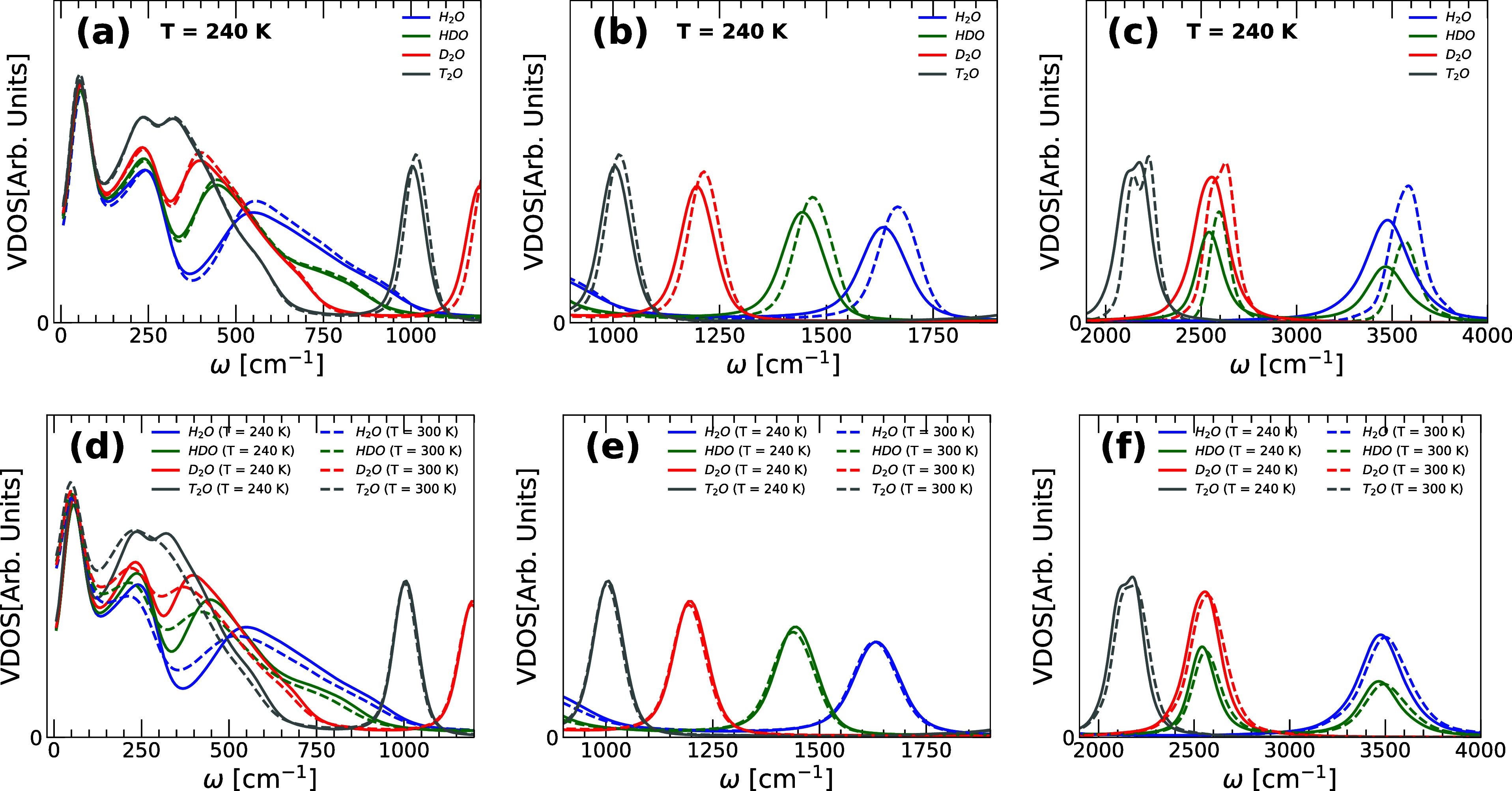
(a–c) Vibrational density of states (VDOS) of H_2_O, HDO, D_2_O, and T_2_O obtained from RPMD (solid lines) and classical MD simulations (dashed lines) of q-TIP4P/F water at *T* = 240 K and *v* = 18.0 cm^3^/mol. The VDOS is shown for different frequency regions corresponding to the (a) translational and librational modes, (b) bending modes, and (c) stretching modes. Increasing the isotope mass shifts the VDOS spectra of water toward lower frequencies. Note the stretching mode frequencies of HDO [green lines in (c)] split into two peaks due to the OH and OD stretching modes (see text). In all cases, the inclusion of NQE (solid vs dashed lines) shifts slightly the (b) bending and (c) stretching modes to lower frequencies, while barely affecting the (a) translational/librational modes. (d–f) VDOS for H_2_O, HDO, D_2_O, and T_2_O from RPMD simulations at *T* = 240 K [solid lines, from (a–c)] and 300 K (dashed lines). Decreasing the temperature shifts the (d) translational/librational modes toward higher frequencies, while leaving the (e) bending and (f) stretching modes practically unaffected.

The stretching mode region of the VDOS of HDO is particularly interesting. Figure 4c shows a bimodal VDOS for HDO with peaks centered at ω_1_ ≈ 2550 cm^–1^ and ω_2_ ≈ 3500 cm^–1^ (green solid line). The peak centered at ω_1_ corresponds to the stretching modes associated with the OD covalent bond. Indeed, the stretching mode peak of D_2_O (solid red line) is centered at ω_1_ as well. Similarly, the HDO VDOS peak centered at ω_2_ corresponds to the stretching modes associated with the OH covalent bond; the stretching mode peak of H_2_O (solid blue line) is also centered at ω_2_. Briefly, the stretching bands of HDO can be interpreted as an equally weighted superposition of the stretching bands of H_2_O and D_2_O.

The VDOS shown in Figure 4a–c are calculated at *T* = 240 K; one may wonder how these VDOS are affected by changes in temperature. To address this question, we show in Figure 4d–f the VDOS of the different water isotopes at *T* = 240 and 300 K (solid and dashed lines, respectively). Briefly, in all cases, the vibrational and stretching bands of the VDOS are barely affected by temperature. The main temperature effects occur in the VDOS translational/librational modes (Figure 4d), which shift toward lower frequencies as the temperature increases. This is consistent with previous studies, which reported a similar behavior in the density of states of the inherent structures for classical q-TIP4P/F and TIP4P/2005 water.^72,73^

For a better comparison with experiments, we also calculate the infrared (IR) spectra of water and its isotopes from our RPMD simulations of q-TIP4P/F water at *T* = 300 K. The IR spectra are obtained by Fourier-transforming the Kubo-transformed dipole moment autocorrelation function;^34,69^ see the SI.

The IR spectra of H_2_O, HDO, D_2_O, and T_2_O are shown in Figure 5 alongside the experimental IR spectra (solid black lines) reported in refs 35, 74 and 75 . For all the isotopes studied, the IR spectra obtained from RPMD simulations are in relatively good agreement with the experiments, including/excluding NQE (solid and dashed lines) leads to minor changes. Overall, the position of the low- and intermediate-frequency peaks, corresponding to the translational/librational and bending modes, respectively, are rather well reproduced by the RPMD/MD simulations. However, differences are noticeable in the position of the high-frequency peaks, corresponding to the stretching modes, of the RPMD/MD simulations and experimental IR spectra. Interestingly, the IR spectra of HDO at high frequencies are bimodal, with one peak associated with the OD stretching modes (ω_1_ ≈ 2550^–1^) and the other with the OH stretching modes (ω_2_ ≈ 3500^–1^). This is fully consistent with the VDOS of HDO shown in Figure 4c.

**Figure 5:**
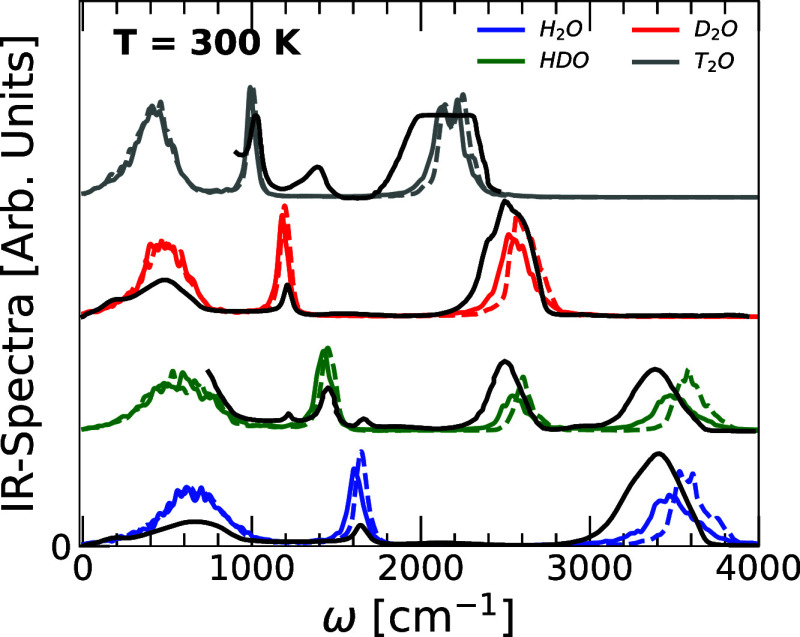
IR spectra of H_2_O, HDO, D_2_O, and T_2_O obtained from RPMD simulations at *T* = 300 K and *v* = 18.00 cm^3^/mol (*P* ≈−25 – 0 MPa, see Figure 2a) using the q-TIP4P/F model (solid lines). The experimental IR-spectra of the water isotopes at *T* = 300 K and *P* = 0.1 MPa are also included from refs;^35^,^74^ and^75^ black lines. The experimental IR spectrum of HDO reported in ref 35 is for a 1:1, H_2_O:D_2_O mixture, which produces a composition of approximately 25% H_2_O, 50% HDO, and 25% D_2_O. This system is comparable but not identical to the HDO system we study using PIMD/RPMD simulations. For comparison, we also include the IR spectra of H_2_O, HDO, D_2_O, and T_2_O obtained from classical MD simulations (dashed lines). In all cases, the IR spectra obtained from the RPMD/MD simulations compare reasonably well with the corresponding experimental IR spectra. The inclusion of NQE leads to minor changes in the IR spectras.

The dynamical properties of the water isotopes studied, including the VDOS and IR spectra shown in Figures 4 and 5, are obtained from RPMD simulations with the PILE thermostat on. This is similar to thermostated RPMD simulations (T-RPMD); however, contrary to the T-RPMD technique, we keep a small friction coefficient (γ = 0.1 ps^–1^) of the PILE thermostat on the zero-frequency mode. As shown in ref 36, our values of *D*(*T*) for H_2_O are identical (within error bars) to the corresponding values of *D*(*T*) obtained using the NVE ensemble (true RPMD) and are consistent with the value of *D* reported in ref 34, at *T* = 300 K; see ref 36. While RPMD-based techniques are reliable to estimate some dynamical properties, such as *D*(*T*), they may induce broadening of the vibrational spectra. Indeed, our spectra in Figure 5, show the correct location of the IR spectra peak of the different water isotopes. However, some of the vibrational peaks appear broadened relative to the experiment (again, a known limitation of the RPMD techniques). Nonetheless, our results indicate the meaningful shift in the IR spectra peaks location due to isotope substitution and are reliable for quantifying isotope-induced shifts.^70,76^ Since our primary focus is on the relative frequency shifts in the VDOS and IR spectra due to NQE, the use of a T-RPMD-like method is well justified. In the future, it would be interesting to apply more advanced techniques such as the temperature-elevation path-integral coarse-graining (Te PIGS) method, which avoids peak broadening and internal mode artifacts while preserving quantum mechanical accuracy.^76,77^

### Structural Properties

#### Radial Distribution Functions

Next, we focus on the oxygen–oxygen (OO), oxygen-X (OX), and X-X radial distribution functions of water, where X refers to hydrogen, deuterium, or tritium. The OO, OX, and XX radial distribution functions (RDFs) of H_2_O, HDO, D_2_O, and T_2_O are shown in Figure 6a–c. Results are from PIMD simulations of q-TIP4P/F water at *v* = 18.0 cm^3^/mol and *T* = 240 K. For comparison, we also include the RDFs for H_2_O obtained from classical molecular dynamics (MD) simulations (black line). The differences in the RDFs among the water isotopes are minor; as the mass of the isotope increases, the peaks of the RDF become slightly more pronounced, indicating that water becomes somewhat more structured. For example, T_2_O (gray line) is slightly more structured than H_2_O (blue line). A comparison of the RDFs for H_2_O from MD and PIMD simulations (blue and black lines) indicates that NQE are also minor (*T* = 240 K); introducing NQE (PIMD simulations) leads to slightly less structured liquids (smaller peaks in the RDFs), consistent with prior computational studies.^34,36,39,42,78^

**Figure 6:**
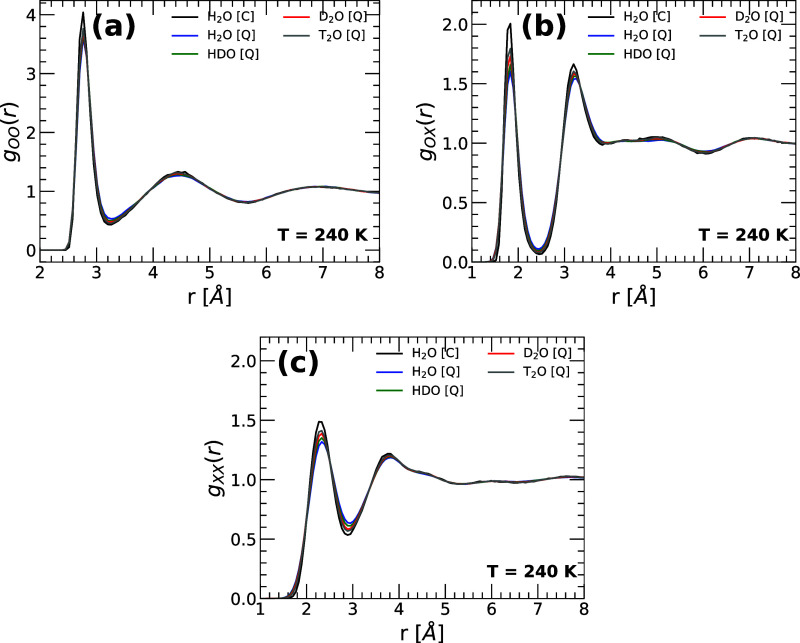
(a) Oxygen–oxygen (OO), (b) oxygen-X (OX), and (c) X-X radial distribution functions of water, where X refers to hydrogen, deuterium, or tritium. Results are from PIMD simulations at *v* = 18.0 cm^3^/mol and *T* = 240 K of q-TIP4P/F water. For comparison, we also include the RDF of H_2_O obtained from classical MD simulations (black lines). Increasing the mass of the isotope of water slightly increases the peaks of the RDFs, leading to a slightly more structured liquid.

The case of HDO is, again, interesting. A close look at Figure 6a–c shows that the OO, OX, and XX RDFs of HDO are between the corresponding RDFs of H_2_O and D_2_O. However, we find that the OH RDF of HDO is practically identical to the OH RDF of H_2_O. Similarly, the OD RDF of HDO is practically identical to the OD RDF of D_2_O. This suggests that the local environment of the O in HDO is an equally weighted linear combination of the local structures of the O atoms in H_2_O and D_2_O.

#### Local Order Parameters

To characterize the local structure of the target water isotopes, we also study the local structure of the target systems using (i) the local order metric ⟨*d*_fs_⟩ defined in ref 79, and (ii) the tetrahedral order parameter ⟨*q*⟩ defined in ref 80. Details for the calculation of ⟨*d*_fs_⟩ and ⟨*q*⟩ are given in ref 38.

(i) Briefly, the local order parameter ⟨*d*_fs_⟩ quantifies, on average, the distance between the first and second hydration shells of the water molecules in the system; for molecules in a low-density domains ⟨*d*_fs_⟩ ≈ 0.1 nm while ⟨*d*_fs_⟩ ≈ 0 for molecules in high-density domains.^38,79^ Figure 7a shows the values of ⟨*d*_fs_⟩ as a function of temperature for H_2_O, HDO, D_2_O, and T_2_O obtained from PIMD simulations at *v* = 18.0 cm^3^/mol using the q-TIP4P/F model. For comparison, the values for H_2_O obtained from classical MD simulations are shown as a black line. As the system undergoes isochoric cooling, ⟨*d*_fs_⟩ increases monotonically for all the isotopes considered. The differences in ⟨*d*_fs_⟩ among the isotopes are rather small but become more pronounced upon cooling. At a given low temperature, ⟨*d*_fs_⟩ increases along the sequence H_2_O­(quantum) → HDO → D_2_O → T_2_O → H_2_O­(classical), i.e., as NQE becomes less pronounced. For example, at low temperatures, the first and second hydration shells of the water molecules are slightly more separated in T_2_O than in H_2_O.

**Figure 7:**
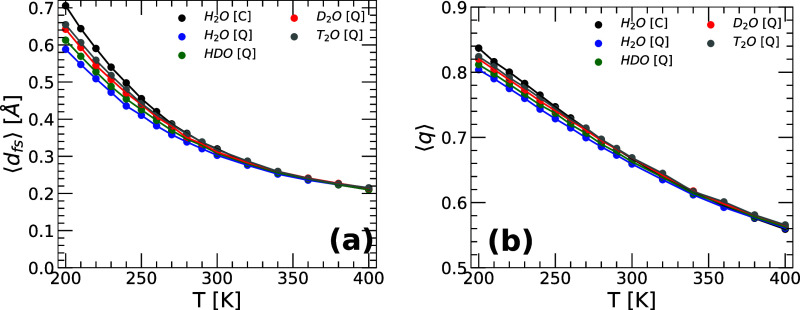
Average local order parameters (a) ⟨*d*_fs_(*T*)⟩ and (b) ⟨*q*(*T*)⟩ as a function of temperature for H_2_O, HDO, D_2_O, and T_2_O. Results are from PIMD simulations of q-TIP4P/F water at *v* = 18.0 cm^3^/mol. For comparison, also included are ⟨*d*_fs_(*T*)⟩ and ⟨*q*(*T*)⟩ of H_2_O obtained from classical MD simulations (black circles). In all cases, ⟨*d*_fs_(*T*)⟩ and ⟨*q*(*T*)> increases monotonically with decreasing temperature, implying that the local environment about the water molecules become, in average, more tetrahedral with increasingly separated first-and second hydration shells. Deviations among the different water isotopes are small and become more pronounced at low temperatures.

(ii) The order parameter ⟨*q*⟩ quantifies, on average, the local tetrahedrality around the water molecules in the system; in a perfect tetrahedral environment, such as in hexagonal ice, ⟨*q*⟩ = 1, while ⟨*q*⟩ = 0 for a system of randomly located molecules/atoms. Figure 7b displays the values of ⟨*q*⟩ as a function of temperature for the same systems included in Figure 7a. Consistent with the behavior of ⟨*d*_fs_(*T*)⟩, ⟨*q*(*T*)⟩ also increases monotonically upon cooling. It follows that, as the first and second hydration shells of the water molecules become more separated, the corresponding local environments of the water molecules become more tetrahedral. These structural changes are slightly more pronounced along the sequence H_2_O­(quantum) → HDO → D_2_O → T_2_O → H_2_O­(classical), i.e., as the atom delocalization (NQE) becomes less pronounced. Nonetheless, the changes in ⟨*q*⟩ among the water isotopes are rather small (<8% at *T* = 200 K). We note that, at high temperatures, the values of ⟨*d*_fs_(*T*)⟩ and ⟨*q*(*T*)⟩ for all the water isotopes practically collapse on one another, indicating that the impact of NQE becomes rather negligible, as expected.

### Hydrogen-Bonding and H/D/T Atom Delocalization Effects

#### Properties of the Hydrogen Bonds

To shed light on the origin of the isotope effects on water discussed above, we next focus on the hydrogen bonds (HB) of H_2_O, HDO, D_2_O, and T_2_O. In the classical MD simulations, we consider that two water molecules form a HB if (i) the corresponding OO distance is *d*_OO_ < 3.5 Å and (ii) the HOO angle is θ_HOO_ < 30 °.^38,81^ Here, we focus on the average OO distance ⟨*d*_OO_^HB^(*T*)⟩ and average HOO angle ⟨θ_HOO_^HB^(*T*)⟩ formed between pairs of hydrogen-bonded water molecules, where ⟨···⟩ indicate an average over time and molecules in the system; the strength/energy of the HB in the different water isotopes is briefly discussed. In the case of PIMD simulations, we apply the same definition of HB given above to molecules within a replica. The values of ⟨*d*_OO_^HB^(*T*)⟩ and ⟨θ_HOO_^HB^(*T*)⟩ are then averaged over all the hydrogen-bonded pairs of water molecules in each replica, and then averaged over all the replicas.^38,82^

Figure 8a,b shows the ⟨*d*_OO_^HB^(*T*)⟩ and ⟨θ_HOO_^HB^(*T*)⟩ of H_2_O, HDO, D_2_O, and T_2_O obtained from PIMD simulations of q-TIP4P/F water at *v* = 18.0 cm^3^/mol (results for H_2_O based on MD simulations are also included, for comparison). In all cases, ⟨*d*_OO_^HB^(*T*)⟩ decreases considerably upon cooling, by ≈0.1 Å for *T* = 400–200 K, even when the volume of the system remains constant (*v* = 18.0 cm^3^/mol). As shown in Figure 8b, ⟨θ_HOO_(*T*)⟩ also decreases monotonically upon cooling. For example, at *T* = 400–200 K, δ⟨θ_HOO_(*T*)⟩ ≈ 3° for all the water isotopes considered (PIMD simulations); δ⟨θ_HOO_(*T*)⟩ ≈ 6° for classical H_2_O (MD simulations). Overall, our results indicate that upon cooling, the water isotopes become more tetrahedral (Figure 7b), the first and second hydration shells of the water molecules become more separated (Figure 7a), and the HB becomes shorter (Figure 8a) and more linear (Figure 8b).

**Figure 8:**
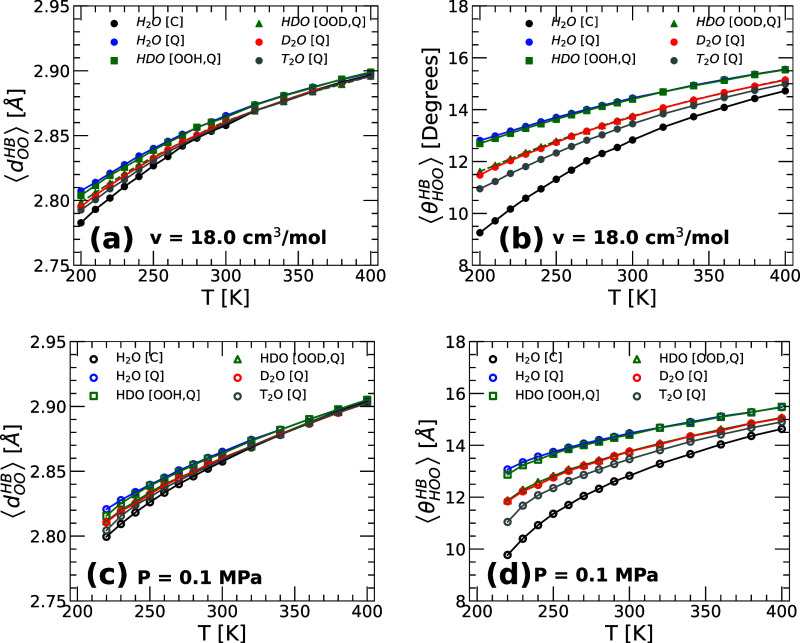
Average (a) OO distance ⟨*d*_OO_^HB^(*T*)⟩ and (b) HOO angle ⟨θ_HOO_^HB^(*T*)⟩ formed between hydrogen-bonded molecules in H_2_O, HDO, D_2_O, and T_2_O. Results are from PIMD simulations at *v* = 18.0 cm^3^/mol using the q-TIP4P/F model. (c,d) Same as (a) and (b) for results obtained from PIMD simulations at *P* = 0.1 MPa. The values obtained from classical MD simulations for H_2_O are also included (black circles). In all cases, as the temperature decreases, the HB become shorter (⟨*d*_OO_^HB^(*T*)⟩ decreases) and more linear (⟨θ_HOO_^HB^(*T*)⟩ decreases). At a fixed temperature, increasing the mass of the water isotope decreases the HB length and the HOO angle; i.e., the HB becomes slightly shorter and more linear along the sequence H_2_O → HDO → D_2_O → T_2_O → H_2_O (classical).

The isotope substitution effects on the HB of water are important. At a given temperature, ⟨*d*_OO_^HB^(*T*)⟩ and ⟨θ_HOO_^HB^(*T*)⟩ decrease monotonically along the sequence H2O → HDO → D2O → T2O, implying that, as the H isotopes become less delocalized, the HB in water becomes shorter and more linear. This supports the view that the *inclusion* of NQE leads to a softer HB-network that is more prone to collapse upon heating and compression, as shown in ref 42. The case of HDO is, again, peculiar. For HDO, we separated the calculation of ⟨*d*_OO_^HB^(*T*)⟩ and ⟨θ_HOO_^HB^(*T*)⟩ based on whether the H or D atom participates in the corresponding HB. The PIMD simulation results for HDO reveal that the ⟨*d*_OO_^HB^(*T*)⟩ and ⟨θ_HOO_^HB^(*T*)⟩ associated with the OH covalent bonds of HDO (green squares) overlap with the ⟨*d*_OO_^HB^(*T*)⟩ and ⟨θ_HOO_^HB^(*T*)⟩ of H_2_O (blue circles). Similarly, the ⟨*d*_OO_^HB^(*T*)⟩ and ⟨θ_HOO_^HB^(*T*)⟩ associated with the OD covalent bonds of HDO (green triangles) overlap with the ⟨*d*_OO_^HB^(*T*)⟩ and ⟨θ_HOO_^HB^(*T*)⟩ of D_2_O (red circles). Our results suggest that the OH covalent bonds in H_2_O and HDO behave identically. Specifically, the corresponding OH RDF (Figure 6b), IR spectra stretching band (Figure 4), and HB geometry (Figure 8) are practically identical in HDO and H_2_O. Similar conclusions apply to the OD covalent bonds of HDO and D_2_O. This suggests that the structural and vibrational properties of the OH and OD covalent bonds in *liquid* water are inherent to the OH/OD covalent bond and rather independent of whether the other covalent bond of the water molecules is an OH or OD covalent bond. We note that while our conclusions are based on MD/PIMD simulations at *v* = 18.0 cm^3^/mol (Figure 8), similar conclusions apply at *P* = 0.1 MPa; see Figure 8c,d.

The different geometries of the HB among the water isotopes suggest that the strength/energy of a HB varies from one water isotope to another. Indeed, the general consensus is that the HB in D_2_O is stronger than in H_2_O,^17^ which is consistent with the slightly higher melting temperature of D_2_O relative to H_2_O (≈4 K^9,17^ ). To estimate the strength of the HB in the water isotopes at a given temperature, we associate an average potential energy for the HB,

$$E_{\mathrm{HB}}(T)≡\frac{E_{\mathrm{pot}}(T)-E_{0}(T)}{n_{\mathrm{HB}}(T)}$$ 3

where *E*_pot_(*T*) is the average potential energy of the (qunatum) system per water molecule, *E*_0_(*T*) is the average potential energy of the corresponding isolated molecule, and *n*_HB_(*T*) is the average number of HB in the system. *E*_HB_(*T*) quantifies the potential energy of an HB after excluding the zero-point energy of the system and (approximately) the internal energy of the water molecule due to thermal fluctuations. Excluding the ZPE is important when comparing the HB strength among the different isotopes since the contributions of the ZPE to the total energy of the system are relevant (see Figure 2b).

Figure 9 shows *E*_HB_(*T*) for all of the water isotopes considered. In all cases, *E*_HB_(*T*) decreases monotonically (becomes increasingly more negative) upon cooling, consistent with the increase in local tetrahedrality and increasingly linear HB formed upon cooling (Figures 7 and 8). While this implies that the HB becomes stronger upon cooling (for all the water isotopes), we note that the changes in the HB energy are rather modest (*T* = 200–400 K). Upon cooling from 400 to 200 K, *E*_HB_(*T*) decreases by δ*E*_HB_ ≈ 0.2–0.5 kJ/mol, depending on the isotope considered. This represents approximately 1.6–3.9% of the HB energy at high temperatures (*T* ≥ 300 K), *E*_HB_ ≈ −12.7 kJ/mol (for classical H_2_O, δ*E*_HB_ ≈ 0.9 kJ/mol, approximately 7.1% of the typical HB energy). Importantly, the observed changes in *E*_HB_ correlate with minor changes in the HB length and angle, as shown in Figure 8.

**Figure 9:**
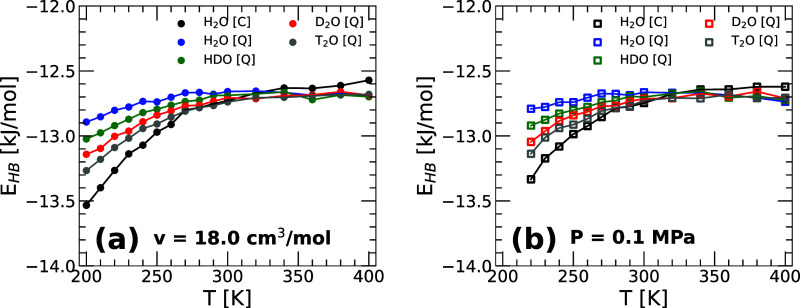
Average potential energy per HB (as defined in eq 3) as a function of temperature for H_2_O, HDO, D_2_O, and T_2_O obtained from PIMD simulations of q-TIP4P/F water. Results are from MD/PIMD simulations at (a) *v* = 18.0 cm^3^/mol and (b) *P* = 0.1 MPa. In all cases, *E*_HB_(*T*) decreases monotonically upon cooling implying that the HB become *slightly* stronger. At a given temperature, *E*_HB_(*T*) decreases along the sequence H_2_O → HDO → D_2_O → T_2_O → H_2_O (classical) suggesting that the HB becomes *slightly* stronger as NQE become less relevant. The same conclusions hold for *P* = 0.1 MPa.

One of the main points of Figure 9 is that at any given temperature, *E*_HB_(*T*) decreases monotonically (becomes more negative) along the sequence H_2_O → T_2_O → D_2_O → HDO → H_2_O (classical). This suggests that as the isotope becomes heavier, and the NQE becomes less relevant, the HB strength increases. However, we note that even at *T* = 200 K, the changes in the HB energy among the water isotopes are rather small. For example, the values for H_2_O (blue line) and T_2_O (gray line) differ by δ*E*_HB_ ≈ 0.4 kJ/mol at *T* = 200 K, representing approximately 3.1% of the HB energy of H_2_O at the same temperature. We note that while the results in Figure 9a are for *v* = 18.0 cm^3^/mol, practically the same results hold at *P* = 0.1 MPa (Figure 9b).

#### Atom Delocalization in the Water Isotopes

The differences in the geometry and strength of the HB among the water isotopes are, ultimately, related to the different degrees of delocalization of the H, D, and T atoms. To quantify the delocalization of the water isotope atoms, we calculated the average radius of gyration *R*_g_(*T*) of the ring polymers associated with the O/H/D/T atoms,

$$R_{g}^{2}=\left\langle \frac{1}{n_{b}}\sum_{k=1}^{n_{b}}({\vec{r}}_{c}-{\vec{r}}_{k}{)}^{2}\right\rangle$$ 4

Here, ${\vec{r}}_{c}$ is the center of mass of the ring-polymers associated with the given atom species, and ${\vec{r}}_{k}$ is the position of the corresponding ring-polymer bead *k* = 1, 2, ···*n*_b_; ⟨···⟩ indicates an average over time and over all ring-polymers (of the same atom type) in the system. In the path-integral formulation of quantum statistical mechanics and hence, in path-integral computer simulations, each atom is represented by a ring-polymer composed of *n*_b_ identical beads, connected by springs with spring constants *k*_sp_ ∝ *T* ^2^.^83,84^ Accordingly, as the temperature decreases, the spring constants also decrease, allowing the beads to spread further apart; physically, the spread of the ring-polymers upon cooling corresponds to the atoms delocalization due to quantum fluctuations. Our PIMD simulations are consistent with this picture. Figure 10b shows the *R*_g_(*T*) for the O, H, D, and T atoms of H_2_O, HDO, D_2_O, and T_2_O. In all cases, *R*_g_(*T*) increases monotonically upon cooling, consistent with previous studies on water and water-like models.^38,85^ Accordingly, all of the atoms become more delocalized as the temperature decreases. Notably, the delocalization of the O atoms is practically identical in all the water isotopes, and hence, it does not depend on the nature of the atoms to which it is covalently bonded (H, D, or T). It also follows from Figure 10b that, at a given temperature, the delocalization increases along the sequence O → T → D → H, i.e., as the mass of the atom decreases (as expected). Note that the delocalization of H in HDO and H_2_O is identical; similarly, the delocalization of D in HDO and D_2_O is identical. Hence, as for the case of the O atoms, the delocalization of the H and D atoms seems to be rather independent of the nature of the water isotope they belong to (under the conditions studied).

**Figure 10:**
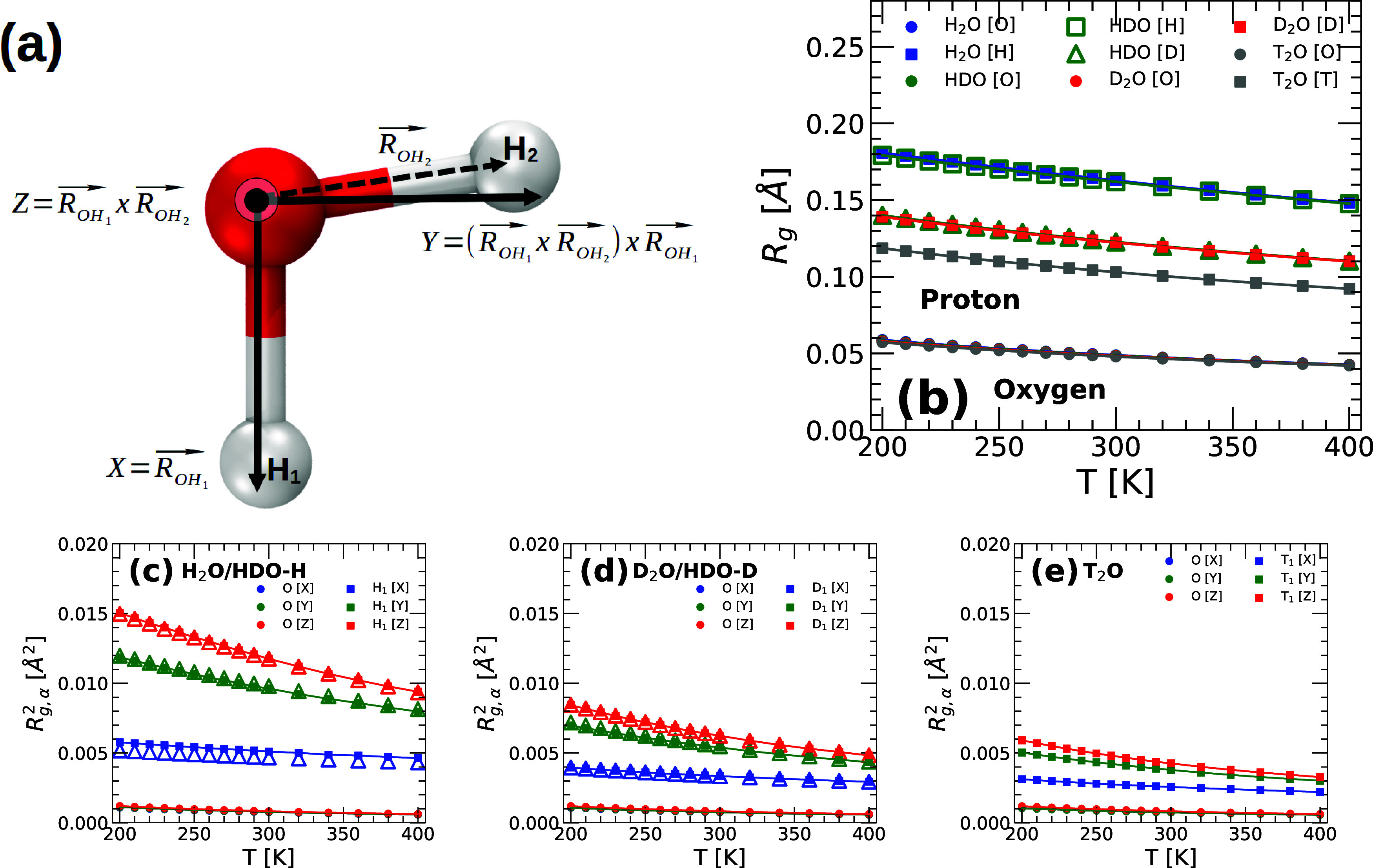
(a) Schematic diagram showing a water molecule with the corresponding (local) reference frame. (b) Radius of gyration, *R*_g_(*T*) of the O, H, D, and T atoms of H_2_O, HDO, D_2_O, and T_2_O obtained from PIMD simulations at *v* = 18.0 cm^3^/mol using the q-TIP4P/F model. Solid circles and squares correspond to the values of *R*_g_(*T*) for the O and H_1_/D_1_/T_1_ atoms of H_2_O, D_2_O, and T_2_O (the data points for O overlap for all the water isotopes studied). The H and D atoms of HDO are represented by green open squares and triangles, respectively. (c) Radius of gyration *R*_g,α_^2^(*T*) along the α = *x*, *y*, and *z* directions for the O and H_1_ atoms of H_2_O (O, solid circles; H, solid squares) and HDO (O, solid circles; H, open triangles). (d) *R*_g,α_^2^(*T*) [α = *x*, *y*, and *z* axis] for the O and D_1_ atoms of D_2_O (O, solid circles; D, solid squares) and HDO (O, solid circles; D, open triangles). (e) *R*_g,α_^2^(*T*) for the O and T_1_ atoms of T_2_O. In all cases, the delocalization of the H_1_/D_1_/T_1_ atoms is preferentially along the direction perpendicular to the O–H_1_/D_1_/T_1_ covalent bond (*z*- and *y*-axis); the delocalization of the O atoms is isotropic (and identical in all cases studied). The delocalization of the H atoms in H_2_O and HDO is practically identical; similarly, the delocalization of the D atoms in D_2_O and HDO is practically identical. The atom delocalization (NQE) is more pronounced along the sequence T → D → H, as expected.

In a previous study, we found that the delocalization of the O atoms in H_2_O for ice I_h_ and LDA at normal pressure was rather isotropic while, instead, the H atoms delocalize preferentially along the directions perpendicular to the corresponding OH covalent bond.^38^ Next, we characterize the anisotropy in the atom delocalization of the water isotopes studied. To do so, for each water molecule, we define a local *xyz*-reference frame as indicated in Figure 10a; the *x*-axis is defined along the O-to-H_1_/D_1_/T_1_ covalent bond while the *y*- and *z*-axis are perpendicular to the O-to-H_1_/D_1_/T_1_ covalent-bond (the water molecule lays on the *x*–*y* plane). We then evaluate the radius of gyration of the ring-polymer associated with the O/H_1_/D_1_/T_1_ atoms along each of these axes,^38^

$$R_{g,\alpha }^{2}=\left\langle \frac{1}{n_{b}}\sum_{i=1}^{n_{b}}({\vec{r}}_{c,\alpha }-{\vec{r}}_{i,\alpha }{)}^{2}\right\rangle$$ 5

where α indicates the corresponding direction, α = *x*, *y*, *z* (see ref 38 for more details about the calculation of *R*_g,α_^2^ based on eq 5). It follows that *R*_g,α_^2^ quantifies the delocalization of the atoms along α = *x*, *y*, and *z*; in addition, *R*_g_^2^ = *R*_g,*x*_^2^ + *R*_g,*y*_^2^ + *R*_g,*z*_^2^.

Figure 10c–e shows the values of *R*_g,*x*_^2^, *R*_g,*y*_^2^, and *R*_g,*z*_^2^ for the O/H_1_/D_1_/T_1_ atoms of the q-TIP4P/F water isotopes studied (as expected, our conclusions do not depend on whether one considers atoms H_2_/D_2_/T_2_ or H_1_/D_1_/T_1_ for the analysis below). In the case of the O atoms, *R*_g,*x*_^2^(*T*) ≈ *R*_g,*y*_^2^(*T*) ≈ *R*_g,*z*_^2^(*T*) at all temperatures, independent of the water isotope considered. Accordingly, consistent with ref 38, the delocalization of the O atoms is isotropic. Instead, the delocalization for H_1_, D_1_, and T_1_ atoms is anisotropic, with *R*_g,*z*_^2^(*T*) > *R*_g,*y*_^2^(*T*) > *R*_g,*x*_^2^(*T*), for H_2_O, D_2_O, and T_2_O; note that the values of *R*_g,*z*_^2^(*T*) and *R*_g,*y*_^2^(*T*) are rather close to one another. Hence, consistent with ref 38, the delocalization of the H/D/T atoms is preferentially along the directions perpendicular to the corresponding covalent bond (*z*- and *y*-directions), slightly more pronounced along the *z*-direction (perpendicular to the HOH plane). Interestingly, the anisotropy in the atoms' delocalization becomes more pronounced along the sequence T → D → H, i.e., as the isotope mass decreases and NQE becomes more pronounced; see Figure 10c–e.

Once again, the case of HDO is peculiar. The triangles in Figure 10c correspond to the *R*_g,α_^2^(*T*) (α = *x*, *y*, *z*) for the H atoms in HDO. The results for H_2_O (squares) and HDO (triangles) in Figure 10c overlap with one another, implying that the anisotropies of the H atoms in H_2_O and HDO are practically identical. Similarly, Figure 10d shows that the *R*_g,α_^2^(*T*) (α = *x*, *y*, *z*) values for the D atoms in HDO (triangles) and D_2_O (squares) are practically identical. Accordingly, the delocalization of the H/D atoms in a water molecule (at the studied conditions) is a property of H/D and hence, it is insensitive to whether there is an H or D atom in the other covalent bond of the given water molecule. Figure 11 shows snapshots of a typical covalent bond of H_2_O, HDO, D_2_O, and T_2_O molecules at *T* = 200 K (*v* = 18.0 cm^3^/mol), where the H/D/T atoms delocalization is largest. The snapshots confirm that the oxygen atom for all isotopes is delocalized isotropically, while the H/D/T exhibit a delocalization that is preferentially along the directions perpendicular to the O-to-H/D/T covalent bond direction.

**Figure 11:**
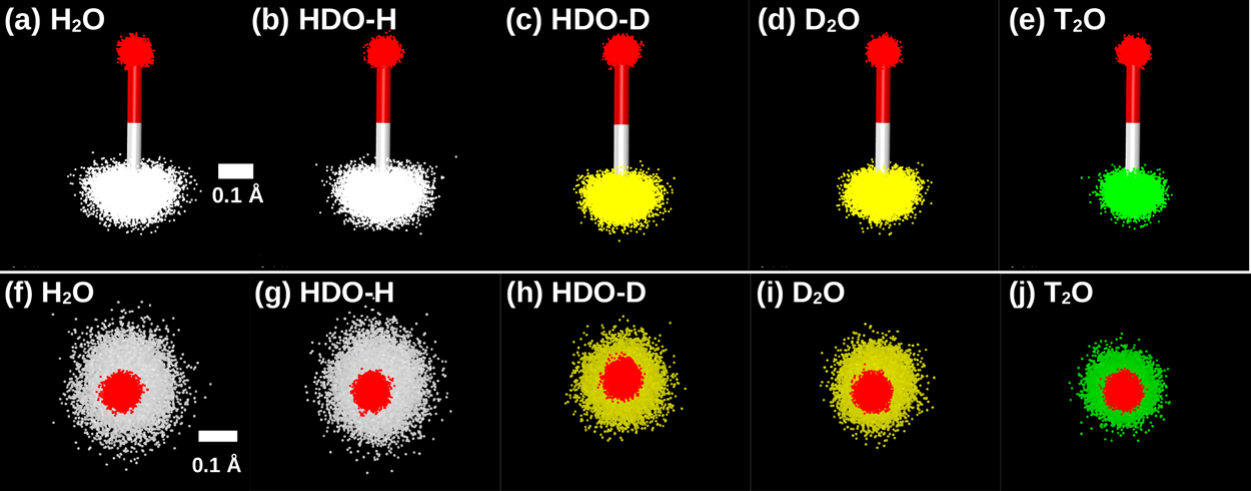
Snapshots showing a covalent bond of the H_2_O, HDO, D_2_O, and T_2_O molecules obtained from PIMD simulations using the q-TIP4P/F model at *T* = 200 K and *v* = 18.0 cm^3^/mol. In (a–e), we show the O atom and only the H_1_/D_1_/T_1_ atom; the view is along the *z*-axis defined in Figure 10a with the molecule lying in the xy-plane. Panels (f–j) are the same covalent bonds included in panels a-e, but the view is along the covalent bond, from the O to the H_1_/D_1_/T_1_ atom. In all snapshots, red and white/yellow/green dots represent the ring-polymer beads of the O and H/D/T atoms, respectively. While each atom/ring-polymer is composed of *n*_b_ = 32 beads, we overlap the beads from all of the molecules in the system (from a single configuration). For the case of HDO (b,c,g,h), the H/D atoms/ring-polymer are shown separately. The same length scale is used in all snapshots (the bar corresponds to 0.1 Å).

## Summary and Discussion

In this work, we perform PIMD simulations of H_2_O, HDO, D_2_O, and T_2_O at both (i) constant volume (*v* = 18.0 cm^3^/mol), and (ii) constant pressure (*P* = 0.1 MPa) across a wide range of temperatures, including the equilibrium and supercooled regimes of water. Our aim is to expose the isotope substitution effects on key (A) thermodynamic, (B) dynamic, and (C) structural properties of water at low pressures (large volumes) and relate these effects to (D) the different atom delocalization (NQE) of the H/D/T isotopes.

### Thermodynamics

Our MD/PIMD simulations of q-TIP4P/F water indicate that some thermodynamic properties, including the molar volume at constant pressure (*P* = 0.1 MPa), the pressure at constant volume (*v* = 18.0 cm^3^/mol; H_2_O density of ρ = 1.0 g/cm^3^), and the isothermal compressibility (volume fluctuations) are weakly affected by isotope substitution effects (Figures 1a–c and 2a). Other properties, including the total energy, enthalpy, and isochoric/isobaric heat capacity, vary considerably with the water isotope considered (Figures 1d,e and 2b,c).

Although our PIMD simulations of q-TIP4P/F water indicate that some thermodynamic properties, particularly the isothermal compressibility κ_*T*_, are weakly sensitive (within error bars) to isotope substitution effects at ambient pressure, this does not imply that NQE values are entirely negligible for such properties. Indeed, isotope substitution effects are expected to be relevant at low temperatures and/or under pressure, e.g., close to the postulated liquid–liquid critical point (LLCP) location (*P*_c_, *T*_c_). Experiments^86^ and PIMD simulations^37^ indicate that the LLCP in H_2_O and D_2_O are located at slightly different pressures and temperatures. Accordingly, the T-dependence of the isothermal compressibility of H_2_O, HDO, D_2_O, and T_2_O must differ as *P* → *P*_c_. Near the LLCP, the impact of NQE on water is expected to be more pronounced, affecting the thermodynamic and dynamical properties;^54^ NQE remain essential in understanding the broader phase behavior of water.

### Dynamics

An important finding of this work is the impact that NQE has on the diffusion coefficients of water isotopes, particularly at low temperatures. As shown in Figure 3, H_2_O diffuses much faster than its heavier isotopes, with the effect becoming more pronounced below *T* ≤ 300 K, where NQE are expected to be most relevant. We note that the slowing down of water with increasing isotope mass is expected even from a classical mechanics point of view. However, classically, one would expect a T-independent isotope substitution effect on the water dynamics. In this regard, the increasing isotope effects on the diffusion coefficients of water (Figure 3) are quantum mechanical in nature. Our MD/RPMD simulations also show that all water isotopes exhibit a dynamical crossover, from an Arrhenius dynamics at high temperatures (*T* > 300 K) to non-Arrhenius behavior (*T* < 300 K). At low temperatures, the dynamics of all water isotopes can be described by MCT (eq 2). Our results are consistent with the interpretation that water’s dynamical strong-to-fragile crossover upon cooling stems from an underlying structural change in water, from a high-density (HDL) to low-density (LDL) liquid as the temperature decreases (see refs 87–89 ). In addition, we note that while MCT captures the fragile dynamics at 200 < *T* < 300 K, it is possible that the dynamics of water become Arrhenius again at lower, cryogenic temperatures. Such a strong-to-fragile-to-strong transition in the dynamics of water is supported by theoretical/computational studies and recent experiments.^37,65,87–91^

Our analysis of the vibrational density of states (VDOS) highlights shifts in the vibrational modes due to isotopic substitution. As expected, the heavier isotopes exhibit lower-frequency vibrational modes, with T_2_O showing the most substantial redshift (Figure 4). These findings from RPMD simulations are consistent with our IR spectra at *T* = 300 K (Figure 5), which are in good agreement with the experimental IR spectra. Overall, these results highlight the importance of incorporating the NQE when studying the vibrational properties of water and its isotopes.

### Structure

The average structure of water exhibits minor changes among the studied water isotopes. Specifically, the RDFs become slightly sharper along the sequence H_2_O → HDO → D_2_O → T_2_O (Figure 6). Accordingly, the heavier the isotope, the (slightly) more structured the liquid water. These structural changes are further reflected in the local order metrics ⟨*d*_fs_⟩ (which quantifies the average separation between the molecules' first and second hydration shells) and ⟨*q*⟩ (which quantifies the molecule's average local tetrahedrality). Indeed, both ⟨*d*_fs_⟩ and ⟨*q*⟩ increase (slightly) along the sequence H_2_O → HDO → D_2_O → T_2_O, particularly at low temperatures (Figure 7). The isotope substitution effects on the RDFs, ⟨*d*_fs_⟩, and ⟨*q*⟩ can be traced down to the isotope effects on the HB. Specifically, our MD/PIMD simulations show that increasing the mass of the isotopes leads to shorter and more linear HBs (Figure 8). To summarize, our structural analysis indicates that, along the sequence H_2_O → HDO → D_2_O → T_2_O, the water isotopes become more tetrahedral (Figure 7b), with the fist and second hydration shells of the water molecules becoming more separated (Figure 7a), and with shorter (Figure 8a) and more linear HBs (Figure 8b). These structural isotope substitution effects become more pronounced at low temperatures.

Of particular interest is how the energy associated with the HB correlates with the structural changes among the water isotopes studied. The shorter and more linear HB along the sequence H_2_O → HDO → D_2_O → T_2_O leads to slightly more energetic (stronger) HB. We note, however, that as for the structural changes, the isotope substitution effects on the HB energy are small. For example, at the lowest temperature considered (*T* = 200 K), the HB energy of T_2_O is only ≈4% lower (more negative; stronger HB) than in H_2_O. While at *T* ≥ 300 K, the HB energy of H_2_O and T_2_O are identical (Figure 9).

### Atom Delocalization

The isotope substitution effects on water’s thermodynamic, dynamic, and, in particular, structural properties are ultimately related to the delocalization of the hydrogen atoms. Our computer simulations indicate that the atom delocalization increases along the sequence T → D → H (as expected). Importantly, the H/D/T delocalization is anisotropic and preferentially along the direction perpendicular to the covalent bond (Figures 10 and 11). It is the subtle differences in the delocalization of the H, D, and T that lead to HB being less linear (and weaker) along the sequence T_2_O → D_2_O → H_2_O. This is consistent with our structural analysis showing that T_2_O is a more structured liquid than H_2_O, with (slightly) more energetic HB, and a stronger HB network (see also ref 42).

The weak NQE reported in this study at 1 bar and *T* = 220 – 400 K is consistent with previous path-integral computer simulation studies.^17,34,92^ It has been proposed that the weak NQE in water are a consequence of two quantum effects that tend to compensate one another, leading to an overall weak NQE at 1 bar and *T* = 300 K.^17^ Specifically, (i) NQE enhances the delocalization of the H/D/T atoms along the corresponding O-to-(H/D/T) covalent bond direction, strengthening the HB; but (ii) NQE also enhances the delocalization of the H/D/T atoms along the directions perpendicular to the corresponding O-to-(H/D/T) covalent bond direction, weakening the HB. Both effects are present in our simulations, but their relative strength changes with the isotope mass and temperature. Our analysis of the radius of gyration along different directions (Figures 10 and 11) reveals that, along the isotope-substitution sequence T_2_O → D_2_O → HDO → H_2_O, atomic delocalization increases for lighter isotopes both along and perpendicular to the covalent bond direction. At high temperatures (*T* ≥ 300 K), these two contributions rather compensate one another, leading to similar hydrogen-bond geometries across the isotopes. However, with decreasing temperatures, the delocalization of the H/D/T atoms along the direction perpendicular to the corresponding O-to-(H/D/T) covalent bond becomes increasingly dominant. The net effect on the energy of the HB varies with temperature and isotope considered; see Figure 9.

## Supplementary Material



## Data Availability

The authors confirm that the data supporting the findings of this study are available within the article.

## Notes

Theory and computational protocol used to calculate the vibrational density of sates (VDOS) and infrared spectra (Figures 4 and 5) from RPMD simulations (PDF)
