# Supplementary material for: Isotope-Substitution Effects on the Thermodynamic, Dynamic, and Structural Properties of Water: H2O, HDO, D2O, and T2O
Source: J Phys Chem B. 2025 Jun 29;129(27):6886–902. doi: 10.1021/acs.jpcb.5c01657 (PMC12257520; doi:10.1021/acs.jpcb.5c01657)
Supplement: Supplementary file 1 [file jp5c01657_si_001.pdf]

Supplementary Material for 'Isotope-Substitution Effects on the Thermodynamic, Dynamic, and Structural Properties of Water: H_2O, HDO, D_2O, and T_2O'

Ali Eltareb[1,2,*], Gustavo E. Lopez[3,4,*], and Nicolas Giovambattista[1,2,3*]

[1]*Department of Physics, Brooklyn College of the City University of New York, Brooklyn, New York 11210, USA*

[2]*Ph.D. Program in Physics, The Graduate Center of the City University of New York, New York, NY 10016, USA*

[3]*Ph.D. Program in Chemistry, The Graduate Center of the City University of New York, New York, NY 10016, USA*

[4]*Department of Chemistry, Lehman College of the City University of New York, Bronx, New York 10468, USA*

[*] aeltareb@gradcenter.cuny.edu, gustavo.lopez1@lehman.cuny.edu, ngiovambattista@brooklyn.cuny.edu

I. CALCULATING THE VIBRATIONAL DENSITY OF STATES FROM RPMD SIMULATIONS

In this appendix, we describe the calculation details for the VDOS shown in Fig. 4 of the main manuscript based on the RPMD approximation to calculate quantum mechanical dynamical properties. In the RPMD approximation, one first evaluates the Kubo-transformed velocity autocorrelation function of the system,

$$\tilde{C}_{v\cdot v}(t) = \frac{1}{N}\sum_{i=1}^{3N} \langle \bar{v}_i(t) \cdot \bar{v}_i(0) \rangle \tag{1}$$

where $3N$ is the number of water atoms and $\bar{v}_i(t)$ is the bead-averaged velocity of ring-polymer i. The VDOS is then obtained by evaluating the Fourier transform of Eq. 1,

$$VDOS(\omega) = \frac{1}{2\pi}\int_{-\infty}^{\infty} e^{-i\omega t}\tilde{C}_{v\cdot v}(t)dt \tag{2}$$

The Kubo-transformed velocity autocorrelation function was calculated using Eq. 1 for up to 2.5 ps from a 5-ps RPMD simulation at constant temperature and volume. To improve the statistics, at each temperature, we also average the Kubo-transformed velocity autocorrelation function over 8 consecutive 5 ps-trajectories. In order to smooth the Kubo-transformed velocity autocorrelation function, a Gaussian of the form $\exp(-\alpha t^2)$ was applied prior to the Fourier transform calculation [1, 2] (the parameter α was set to 1.0 ps^{-2}).

II. CALCULATING THE INFRARED SPECTRA FROM RPMD SIMULATIONS

In this section, we explain the calculation details for the IR spectra shown in Fig. 5 of the main manuscript based on the RPMD approximation to calculate quantum mechanical dynamical properties. Within the RPMD approximation, one first evaluates the Kubo-transformed dipole moment autocorrelation function [3, 4]

$$\tilde{C}_{\boldsymbol{\mu}\cdot\boldsymbol{\mu}}(t) = \left\langle \sum_{i=1}^{N} \bar{\boldsymbol{\mu}}_{\boldsymbol{i}}(t) \cdot \bar{\boldsymbol{\mu}}_{\boldsymbol{i}}(0) \right\rangle \tag{3}$$

where $\bar{\boldsymbol{\mu}}_{\boldsymbol{i}}$ is the bead-averaged dipole moment of ring-polymer i

$$\bar{\boldsymbol{\mu}}_{\boldsymbol{i}} = \frac{1}{n_b}\sum_{k=1}^{n_b} \boldsymbol{\mu}_{\boldsymbol{k,i}} \tag{4}$$

where $\boldsymbol{\mu_{k,i}}$ is the dipole moment of water molecule i belonging to replica k. The IR spectra is then obtained by evaluating the Fourier transform of Eq. 3,

$$I(\omega) \propto \int_{-\infty}^{\infty} \tilde{C}_{\mu\cdot\mu}(t) e^{-i\omega t} dt \tag{5}$$

where ω is the vibrational frequency.

The IR spectra can also be computed using the Kubo-transformed dipole moment time derivative autocorrelation function [1, 5]

$$\tilde{C}_{\dot{\mu}\cdot\dot{\mu}}(t) = \left\langle \sum_{i=1}^{N} \dot{\boldsymbol{\mu}}_{\boldsymbol{i}}(t) \cdot \dot{\boldsymbol{\mu}}_{\boldsymbol{i}}(0) \right\rangle \tag{6}$$

where $\dot{\boldsymbol{\mu}}_{\boldsymbol{i}}$ is the time derivative of Eq. 4. The IR spectra is then given as

$$I(\omega) \propto \int_{-\infty}^{\infty} \tilde{C}_{\dot{\mu}\cdot\dot{\mu}}(t) e^{-i\omega t} dt \tag{7}$$

In this work, we calculate $I(\omega)$ using Eqs. 6 and 7. The Kubo-transformed autocorrelation function in Eq. 6 is computed from a 10 ps-long RPMD simulation at constant temperature and volume. To improve our results we average over 5 consecutive 20 ps-long trajectories. Before taking the Fourier transform in Eq. 7, a Gaussian of the form $\exp(-\alpha t^2)$ is applied to the autocorrelation function (with $\alpha = 2.0$ ps^{-2}), in order to have the autocorrelation function smoothly decrease to 0.

[1] M. Praprotnik, D. Janežic, and J. Mavri, J. Phys. Chem. A **108**, 11056 (2004).

[2] M. Wilson, P. A. Madden, M. Hemmati, and C. A. Angell, Phys. Rev. Lett. **77**, 4023 (1996).

[3] S. Habershon, T. E. Markland, and D. E. Manolopoulos, J. Chem. Phys. **131**, 024501 (2009).

[4] S. Habershon, D. E. Manolopoulos, T. E. Markland, and T. F. Miller III, Annu. Rev. Phys. Chem. **64**, 387 (2013).

[5] M. Rossi, H. Liu, F. Paesani, J. Bowman, and M. Ceriotti, J. Chem. Phys. **141** (2014).
